# Prevalence of hip dislocation among children with cerebral palsy in regions with and without a surveillance programme: a cross sectional study in Sweden and Norway

**DOI:** 10.1186/1471-2474-12-284

**Published:** 2011-12-16

**Authors:** Areej I Elkamil, Guro L Andersen, Gunnar Hägglund, Torarin Lamvik, Jon Skranes, Torstein Vik

**Affiliations:** 1 Department of Laboratory Medicine, Children's and Women's Health, Faculty of Medicine, Norwegian University of Science and Technology, Trondheim, Norway; 2 Department of Paediatrics, St. Olav's University Hospital, Trondheim, Norway; 3 The Norwegian Cerebral Palsy Register, Habilitation centre, Vestfold Hospital Trust, Tønsberg, Norway; 4 Department of Orthopaedic surgery, Lund University, Lund, Sweden; 5 Department of Orthopaedic surgery, St. Olav's University Hospital, Trondheim, Norway

## Abstract

**Background:**

Hip dislocation is a serious complication among children with cerebral palsy (CP). The aim of this study was to compare the prevalence of hip dislocation among children with CP in an area providing regular care with an area providing hip surveillance services.

**Methods:**

This is a cross-sectional study in seven Norwegian counties providing regular care and one Swedish healthcare region where a hip surveillance programme was introduced in 1994. Data were provided by the Norwegian Cerebral Palsy Register and the CP Register in Southern Sweden. Children born 1996 - 2003 with moderate to severe CP, defined as Gross Motor Classification System (GMFCS) levels III - V, were included. In all, 119 Norwegian and 136 Swedish children fulfilled the criteria. In Norway, data on hip operations and radiographs of the hips were collected from medical records, while these data are collected routinely in the Swedish register. The hip migration percentage was measured on the recent radiographs. Hip dislocation was defined as a migration percent of 100%.

**Results:**

The proportion of children at GMFCS levels III - V was 34% in the Norwegian and 38% in the Swedish population. In the Norwegian population, hip dislocation was diagnosed in 18 children (15.1%; CI: 9.8 - 22.6) compared with only one child (0.7%; 95% CI: 0.01 - 4.0) in Southern Sweden (p = < 0.001). Hip surgery was performed in 53 (44.5%) of the Norwegian children and in 43 (32%) of the Swedish children (p = 0.03). The total number of hip operations was 65 in Norway and 63 in Sweden. Norwegian children were first operated at a mean age of 7.6 years (SD: 2.9) compared with 5.7 years (SD: 2.3) in Sweden (p = 0.001).

**Conclusions:**

The surveillance programme reduced the number of hip dislocations and the proportion of children undergoing hip surgery was lower. However, with the surveillance programme the first operation was performed at a younger age. Our results strongly support the effectiveness of a specifically designed follow-up programme for the prevention of hip dislocation in children with CP.

## Background

Dislocation of the hip among children with cerebral palsy (CP) is a severe complication following insidious hip displacement. Chronic increase in muscle tone, muscle power imbalance and fixed contractures around the hip joint together with unbalanced posture contribute to the progressive displacement of the femoral head out of the acetabular socket [1-4]. The prevalence of total hip dislocation is reported to be 10 - 15% [5-7], while hip displacement to various degrees is estimated to occur in 25 - 60% [3,8,9]. The risk of dislocation is increasing with increased gross motor disability with the highest risk among children at level five classified by the Gross Motor Function Classification System (GMFCS)[10,11].

Hip dislocation is preventable, but detection of a hip at risk must be based on both clinical and radiological examinations [7,9,12]. In Southern Sweden, a follow-up programme for CP was started in 1994 with a specific aim to identify patients at risk for hip dislocation and to prevent this complication. During the following ten years hip dislocation dropped from 8% to 0.5% [6]. Comparable results were reported from an Australian programme introduced in 1997 [13]. However, during the years when these follow-up programmes were implemented, treatment of spasticity has dramatically improved in particular through the introduction of botulinum neurotoxin (BoNT) and intrathecal baclofen [14,15]. Together with increased awareness of the risk of hip displacement, these new treatment modalities might have resulted in reduction of hip displacement even in centres where the surveillance programme had not been implemented.

Ideally the efficacy of a new treatment option should be documented by randomised controlled trials (RCT). However, in CP, comprising a heterogeneous group of patients, conducting RCTs is difficult. A different approach may, therefore, be to compare the outcome of treatment in different treatment centres with otherwise comparable quality of care, so called practice-based evidence. Adopted in some fields of medicine, it is proved to be useful in real life mainly due to the possibility of comparing heterogeneous groups and including even severely affected patients in contrast to RCTs with restricted inclusion criteria [16]. An example of a similar approach was used in the improvement in treatment of children with acute lymphocytic leukaemia in the early 1980s [17].

The medical services and the level of care for children with CP in the Scandinavian countries are quite similar [18]. The aim of this study was, therefore, to compare the prevalence of hip dislocation in a Norwegian population receiving regular care with a group of children in Southern Sweden included in the hip surveillance programme, and to compare the number and type of hip operations performed in these areas. Our main hypothesis was that the prevalence of dislocation would be higher in the areas providing regular care compared with the area providing the surveillance services. Since radiological screening from early childhood is an essential feature of the surveillance, resulting in the early detection of candidates for preventive surgery, we also hypothesised that the proportion of children operated, and the number of hip operations, would be higher in the surveillance area.

## Materials and methods

This is a cross-sectional study in seven Norwegian counties (total population: 2.0 million) and one Swedish healthcare region (total population 1.4 million). The population in the studied Norwegian counties corresponds to 41% of the total population in Norway, while in Sweden the studied background population comprises 15% of the total population. Eligible for this study were children born 1996 - 2003 with moderate to severe CP estimated as GMFCS levels III - V.

In the Norwegian counties, all children with moderate to severe CP visit a paediatric rehabilitation centre, at least once a year. At these visits one main focus is on motor function, spasticity treatment and the potential need for surgery. However, for children born during 2003 and earlier there were no common guidelines to identify children at risk for hip dislocations or for early preventive surgery. Thus, the seven Norwegian counties are in this study entitled the 'regular care areas'. In Southern Sweden, a follow up programme to prevent hip dislocation was started in 1994 and this area is entitled the 'surveillance area'. The programme includes annual radiological examination of children at GMFCS levels III - V as soon as the diagnosis has been confirmed until the age of eight years. Above the age of eight, the frequency of x-rays is individually evaluated based on the results of earlier examinations. Children at risk for hip dislocation are treated with early preventive surgery. A similar surveillance programme has been successively introduced in Norway since 2006, covering whole Norway since 2010.

In Norway, data on hip displacement, orthopaedic surgery, x-ray examinations and detailed spasticity treatment were collected retrospectively for this study from medical records in 2010, while other clinical data were obtained from the Norwegian Cerebral Palsy Register. In Southern Sweden all data were collected prospectively (details of the two registers have been published) [10,19,20].

According to the Surveillance of CP in Europe (SCPE) definition, *cerebral palsy *is a "group of permanent and non-progressive disorders of movement and posture caused by a central nervous lesion, damage or dysfunction originating early in life" [21]. This definition, as well as the classification of CP subtypes recommended by SCPE, was used by the Norwegian and Swedish CP registers.

*Gross motor function *of each child was estimated by the treating rehabilitation centre and classified in the CP registers into five levels according to the GMFCS, where level I has the least and level V has the most severe motor impairment [19,22,23].

In the Norwegian counties there were 494 children with CP born between 1^st ^January 1996 and 31^st ^December 2003, corresponding to a prevalence of 2.65 per 1000 live births. Detailed clinical data were available on 357 of these children. The primary reason for the missing detailed data was work overload for local doctors. Only one family refused to participate in the CP-register. We included all children who lived in the area during the period 1996 to 2010 and those who died before 1^st ^January 2011 (n = 12). Of the 357 children with detailed clinical data, 121 (33.9%) were classified as GMFCS level III -V. One child moved to the area at the age of five with partially displaced hip and was excluded and one patient refused to participate in the hip study. In Southern Sweden there were 358 children with CP with the same inclusion criteria, corresponding to a prevalence of 2.70 per 1000 live births. Of these children, 136 (38%) were classified as GMFCS level III - V. Thus, 119 Norwegian and 136 Swedish children fulfilled the inclusion criteria and their data were further analysed.

The main outcome variable was *hip dislocation*. Anonymous copies of the most recent, or preoperative, anteroposterior (AP) radiographs of the pelvis were evaluated by one of the authors (GH). The migration percentage of Reimers (MP) [24] was measured and was compared with the MP values in the Swedish material. In hips with a *Gothic arch *formation of the lateral margin, the midpoint of the arch was used as a reference point according to Cooke *et al*. [5]. Hip dislocation was defined as MP 100% measured on AP pelvic radiographs except in one case where complete bilateral dislocation was diagnosed by computerised tomography (CT) scanning. Three Norwegian children never had radiological hip examination. In another three children we were not able to get a copy of the most recent pelvic radiograph, but the hips were described as normal in the radiologists' reports.

The secondary outcome measure, *hip surgery*, was classified into soft tissue surgery (adductor- and iliopsoas muscle lengthening or release), varus derotation osteotomy of the proximal femur, pelvic osteotomy and femoral head resection respectively. In the Swedish population soft tissue operations always included myotenotomy of adductor longus, gracilis and iliopsoas, while in the Norwegian population gracilis and iliopsoas muscles were not always included in the procedure. Other variables used in the study were age, sex and other modes of spasticity treatment, namely BoNT, intrathecal baclofen (ITB) and selective dorsal rhizotomy (SDR).

### Ethics

In Norway, written informed consent had been obtained from parents to record detailed clinical data in the Norwegian CP register as required by the Regional Ethics Committee (REC) for medical research in the healthcare region of Mid-Norway (reference number: 046-02.2002). The present study was approved by the same ethics board (reference number: 2009/984-2). For this study, parents were informed about the additional collection of data from the medical records and were given the opportunity to decline. The analysis of data from the CP-register in southern Sweden was approved by the Medical Research Ethics Committee at Lund University (LU-443-99). This study was carried out in compliance with Helsinki Declaration.

### Statistical analyses

Chi squared statistics was used to assess differences in proportions between the two study areas, while the difference in mean age at first operation was analysed by independent t-test.

## Results

In all, 119 Norwegian and 136 Swedish children fulfilled the inclusion criteria and their data were further analysed. There were no significant differences between the Norwegian and Swedish study populations in terms of CP prevalence, GMFCS levels and gender (Table 1). The proportion of children classified as dyskinetic CP was, however, higher in Sweden compared with Norway where the proportion with bilateral spastic subtype was higher (Table 1).

**Table 1:** Distribution of CP-subtype according to SCPE, GMFCS level and sex in the two study populations

		Norway		Sweden		
		**n**	**(%)**	**n**	**(%)**	

		**119**	**(100%)**	**136**	**(100%)**	*p*-value

**CP -subtype**						0.002
	Unclassified	4	(3.4)	11	(8.1)	
	Unilateral	2	(1.7)	2	(1.5)	
	Bilateral	86	(72.3)	65	(47.8)	
	Dyskinetic	24	(20.2)	48	(35.3)	
	Ataxic	3	(2.5)	10	7.4	
**GMFCS**						0.74
	Level III	28	(23.5)	36	(26.5)	
	Level IV	54	(45.4)	55	(40.4)	
	Level V	37	(31.1)	45	(33.1)	
**Sex**						0.42
	Boys	73	(61.3)	74	(54.4)	
	Girls	46	(38.7)	62	(45.6)	

GMFCS, Gross Motor Function Classification System.

Hip dislocation (MP 100%) occurred in 18 children (15.1%; CI: 9.8 - 22.6) of the Norwegian population; in nine of them the hips were bilaterally dislocated. Of the 27 dislocated hips, 12 were right and 15 were left sided. In contrast, in Sweden only one child (0.7%; CI: 0.13 - 4.0), considered to be inoperable, had a dislocated hip (p < 0.001 versus the Norwegian population).

The number of children operated and the type of hip surgery are presented in Table 2. The number of children operated was 53 (44%; CI: 35.6 - 53.1) in Norway and 43 (32%; CI: 24.4 - 39.8) in Sweden (p = 0.03). In the Norwegian population a higher proportion of children underwent merely soft tissue release (Table 2). Seven of the children with complete hip dislocation did not undergo hip surgery, three of them died and four were considered to be inoperable. Nine children underwent hip surgery once (five had adductor myotenotomy including psoas in three cases, one had pelvic osteotomy and three patients had femoral head resection). Two children were operated on twice (adductor tenotomy followed by pelvic osteotomy in one case and by femoral head resection in the other).

**Table 2:** Hip surgery (number of patients)

		Norway		Sweden		
		**n**	**(%)**	**n**	**(%)**	***p-*value**

Hip surgery						
	Soft tissue surgery	28	(23.5)	16	(11.8)	0.013
	Femur osteotomy	11	(9.2)	21	(15.4)	0.137
	Pelvic osteotomy	10	(8.4)	6	(4.4)	0.191
	Femoral head resection	4	(3.4)	0	(0.0)	0.046
Total operated		53	(44.5)	43	(31.6)	0.034
Not operated		66	(55.5)	93	(68.4)	
Total		119	(100.0)	136	(100.0)	

The total number and the type of hip operations performed in the two regions are presented in table 3. The number of hip operations carried out was 65 in the Norwegian counties and 63 in Southern Sweden.

**Table 3:** Hip surgery (number of operations)

	Norway			Sweden			
	**n**	**(%)**	**Operation/patient**	**n**	**(%)**	**Operation/patient**	***p*-value**

Soft tissue surgery	36	(55.4)	1.3	28	(44.4)	1.8	0.130
Femur osteotomy	13	(20.0)	1.2	29	(46.0)	1.4	0.002
Pelvic osteotomy	12	(15.6)	1.2	6	(9.5)	1.0	0.147
Femoral head resection	4	(6.2)	1.0	0	(0.0)		0.119
Total operations	65	(100.0)		63	(100.0)		

**Figure 1:**
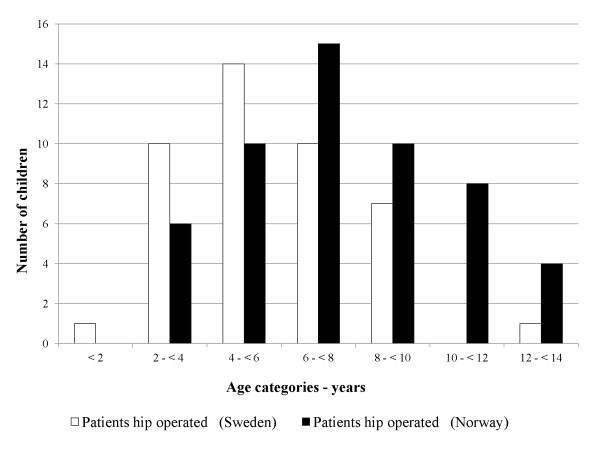
**Age at first hip operation**. Number of children (Y-axis) and age (X-axis) at first hip operation in the Norwegian providing regular care (black bars) and the Swedish population offering a surveillance programme (white bars). Age was grouped in seven categories.

Children in the surveillance area were operated at an earlier age compared with areas providing regular care in Norway (Figure 1). Mean age at first operation was 5.7 years (SD: 2.3) in Sweden and 7.6 years (SD: 2.9) in Norway (p = 0.001).

Spasticity reducing treatment with BoNT and ITB was more common in the Norwegian study population than in the Swedish population, whereas SDR was more common in Southern Sweden (Table 4).

**Table 4:** Spasticity treatment (number and percent of children treated)

		Norway		Sweden		
		**N**	**(%)**	**N**	**(%)**	***p*-value**

Botulinum neurotoxin		78	(65.0)	66	(48.5)	0.008
	Adductors	55	(45.8)	35	(25.7)	0.001
	Iliopsoas	13	(10.8)	0	(0.0)	< 0.001
Baclofen pumpe		29	(24.2)	7	(5.2)	< 0.001
Selective dorsal rhizotomy		1	(0.8)	6	(4.4)	0.08

The mean number of plain anteroposterior x-rays of the pelvis in Sweden was 9 per patient. In Norway, we are only able to account for the mean number of x-rays in two counties. In these two counties, covering 28 of the children, the mean number of plain pelvic x-rays per patient was five. However, four pelvic CT scans had been taken in three of these 28 children. In contrast, no pelvic CT scans had been performed in any of the Swedish children.

## Discussion

We found, consistent with our main hypothesis, that the prevalence of hip dislocation was higher in the Norwegian counties providing regular care compared with Southern Sweden providing hip surveillance services. In contrast to our second hypothesis, we found that the surveillance programme did not lead to an increase in the total number of hip operations. However, children in the surveillance programme were operated at an earlier age than children offered regular care.

The difference in prevalence of hip dislocation and operations between the two regions is unlikely to be due to chance as suggested by the low *p*-values. A strength of the present study is the comparison with contemporary rather than historical data, as has been the case in other studies [6,13]. Thus, our results indicate that a general improvement in treatment of spasticity and increased awareness of hip dislocation are unlikely to explain the effect of the surveillance programme. Another strength is that all pelvic radiographs in this study were evaluated by one investigator, since a marked interrater variation in measurements of the MP has been reported [25,26]. Moreover, the primary clinical data on CP diagnosis, subtype, GMFCS and associated impairments were collected prospectively. However, in the areas providing regular care, data on hip status and surgery had to be collected retrospectively while this was done prospectively in the surveillance programme. Furthermore, in the surveillance area, more children (84%) had a recent (in the last two years) pelvic x-ray than children in the regular care area (73%); a reasonable effect of the programme (data not shown). Thus, it is theoretically possible that a case of hip dislocation or an operation might have been missed among patients offered regular care. This potential misclassification would, however, lead to an underestimation of hip dislocations and operations in the regular care areas. A potential limitation could be that the reviewer of the radiographs was not blinded to area. However, hip dislocations in the regular care area were originally diagnosed as completely dislocated by local clinicians, and no additional hips were deemed dislocated by the reviewer of the present study. Regarding the Swedish population, the radiographs were evaluated prospectively, and before the present study was planned. Since we used a migration percentage of 100 as a cut-off for hip dislocation, it is most unlikely that a complete dislocation had been overlooked.

Another potential limitation is the lack of complete data on 27% of children with CP in the seven Norwegian counties compared with nearly 100% coverage in Southern Sweden. The primary reason for the missing data in Norway is actually work overload on local doctors who were not able to complete the CP-registration forms. Only one family actively refused to participate in the CP-register, and one further family did not want to participate in the present study. If a more severely affected study population had been selected in Norway, this might have contributed to a higher proportion of hip dislocations. However, we have provided evidence that cases included in the Norwegian CP register are likely to be representative of the total CP population in a previous study [27]. Moreover, the distribution of children within GMFCS levels III - V among all children with CP was nearly identical in the two areas.

In contrast to the similar distribution of GMFCS levels in the two study populations, a higher proportion of children were classified as dyskinetic in Sweden compared with Norway, whereas more children were classified as bilateral spastic CP in the Norwegian counties. Previous investigators showed a considerable variation in prevalence of the dyskinetic subtype among European countries and attributed that to differences in the classification of CP [20,28]. However, dyskinetic CP also have increased risk of hip displacement [5,10], and several studies have shown that GMFCS levels are more correlated to hip displacement than the topographic CP subtypes [10,11,29]. Thus, taken together, misclassification or selection bias are unlikely to explain our results.

A potential confounder in this study is that the use of botulinum neurotoxin and baclofen was more common in Norway, since it could be expected to reduce the incidence of hip dislocation [30,31]. However, if this was the case, it would have reduced the differences between the groups. On the other hand, one could speculate if this treatment had masked hip displacement by reducing spasticity and alleviating pain [32-38].

We are not aware of other studies using the same approach to study the effectiveness of a hip surveillance programme to prevent hip dislocations. Our results are, however, consistent with the studies showing a reduction in hip dislocation in the total CP population from 8% to nearly 0% in Southern Sweden and elimination of the need for salvage operations in Australia after commencement of their screening programmes [6,13].

Our results suggest that systematic clinical and radiological follow-up from an early age, as provided by the hip surveillance programme, lead to early detection of hip displacement signified by surgical intervention at a younger age. Nonetheless, the number of children operated on and the total number of operations was not higher in the surveillance compared with the regular care areas. This may be somewhat in contrast to the increase in preventive surgery which was reported in Australia after introduction of a similar screening programme [13].

In the regular care area 23.5% of the children were treated with solely soft tissue release which might have been insufficient to prevent dislocation in some cases [39-41]. In the surveillance area a higher proportion underwent femur osteotomy compared with the regular care areas, and also compared with the Australian surveillance programme. The latter difference is probably due to a longer observation time in Sweden [6,13]. In Southern Sweden a standardised protocol is used for hip surgery as outlined in the CP follow-up programme (CPUP) with early soft tissue surgery followed by femur osteotomy in progressive cases [1], and this most probably explains the variation in types of hip operations in the two study populations.

The difference in prevalence of hip dislocation between the two study populations could theoretically be due to an extraordinary high prevalence in the regular care areas, or an extremely low prevalence in the surveillance area, or both. However, the prevalence of hip dislocation among children with GMFCS III - V in the regular care area corresponds to an estimated prevalence of 5.1% in the total CP-population in this area. This is lower than the 8% reported in Sweden before the surveillance programme was introduced and the 14% reported by Scrutton [9] from South East Thames. Thus, it is unlikely that the prevalence of hip dislocation is extraordinary high in the seven Norwegian counties compared to other areas without a surveillance programme. In contrast, the prevalence of 0.7% among children with GMFCS III - V in the surveillance area in this study is extremely low, and may not be obtained by other centres adopting the programme. Nonetheless, even a seven-fold higher prevalence (4.7% CI: 2.1 - 9.7) would be considerably lower than in the area providing regular care (15.1%; CI: 9.8 - 22.6). Thus, it is likely that the implementation of the surveillance programme in other populations will result in significant reduction in the proportion of children with hip dislocation.

Our results suggested that plain pelvic x-rays were taken more frequently in the surveillance area compared with the area providing regular care. Although we only had data from two counties in the regular care area, this might be a reasonable finding which could be considered a potential "downside" of the surveillance programme. On the other hand, our limited information suggested that the higher number of x-rays per child in the surveillance area partly was outweighed by an apparent need for a pelvic CT scan in a few patients in the regular care area.

## Conclusions

Our results suggest that systematic follow up, including regular radiographic examinations of the pelvis from an early age, followed by early surgery is essential in preventing hip dislocation among children with severe CP.

## Competing interests

The authors declare that they have no competing interests.

## Authors' contributions

AE, TV, GH and GLA designed the study. Data were collected by AE, GLA and JS. Data analysis was carried out by AE supervised by TV. AE made the first draft, then actively improved by all authors.

## Authors' information

Professors TV, JS and Dr. GLA are paediatricians. AE is paediatric registrar. Professor GH and Dr. TL are orthopaedic surgeons.

## Pre-publication history

The pre-publication history for this paper can be accessed here:

http://www.biomedcentral.com/1471-2474/12/284/prepub
